# Successful laparoscopic resection for giant mature cystic teratoma of the pancreas: a case report and surgical refinements

**DOI:** 10.1186/s40792-024-01918-6

**Published:** 2024-05-09

**Authors:** Mayuko Kori, Masashi Tsunematsu, Ran Yao, Ryoga Hamura, Atsushi Yoda, Hidetoshi Endo, Takashi Horiuchi, Kyohei Abe, Takeshi Hisa, Shinji Onda

**Affiliations:** 1 Department of Digestive Surgery, Saku Central Hospital Advanced Care Center, 3400-28, Nakagomi, Saku, Nagano, 385-0051 Japan; 2 Department of Gastroenterology, Saku Central Hospital Advanced Care Center, 3400-28, Nakagomi, Saku, Nagano, 385-0051 Japan

**Keywords:** Dermoid cyst, Laparoscopic distal pancreatectomy, Mature cystic teratoma, Pancreas

## Abstract

**Background:**

Mature cystic teratomas or dermoid cysts of the pancreas complicate surgical approaches because of their anatomical position and ever-growing size. Herein, we report a case of a giant mature cystic teratoma of the pancreas that was successfully resected via complete laparoscopic distal pancreatectomy (LDP).

**Case presentation:**

A 39-year-old female patient was referred to our hospital for the evaluation of a pancreatic tumor. Three years of follow-up revealed that the tumor had increased in size to 18 cm, with hyperintense solid components on diffusion-weighted magnetic resonance imaging. Considering the possibility of malignancy, we decided to perform an LDP. The capsule appeared solid enough to withstand the retraction of the endoscopic forceps. Tumor size made it difficult to dissect the dorsal side of the tumor from the caudal to the cranial side. Early transection of the pancreas and additional ports facilitated dissection of the dorsal side of the tumor. We completed the LDP without intraoperative cyst rupture. On pathological examination, the tumor was diagnosed as a mature cystic teratoma originating from the pancreatic tail. The patient was discharged on postoperative day 13 with no complications.

**Conclusion:**

LDP may be an option for surgical procedures in patients with large cystic lesions of the pancreatic body or tail. Intraoperative observation of the tumor and surgical refinement are necessary to complete the laparoscopic procedure without tumor rupture.

## Background

Mature cystic teratomas or dermoid cysts of the pancreas are very rare, with limited cases reported in the literature, and they show various clinical features in terms of size and tissue components [1–4]. Complete surgical removal is the mainstay of its treatment [5]. The tumor poses a diagnostic challenge and complicates the surgical approach, not only because of its anatomic position but also because of its ever-growing size. Here, we describe the case of a patient with a giant mature cystic teratoma compressing the left renal vein, which was successfully resected by laparoscopic distal pancreatectomy (LDP) without intraoperative tumor rupture.

## Case presentation

**Fig. 1:**
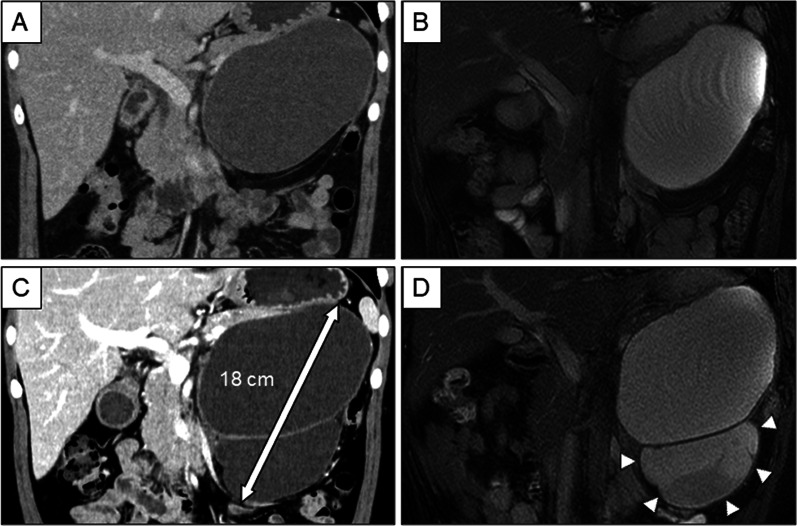
Imaging of pancreas tumor. **A** Contrast-enhanced computed tomography revealed a 14-cm, well-demarcated cyst. **B** Magnetic resonance imaging was remarkable for a smooth mass with homogeneous content at the pancreatic tail. **C** The mass had enlarged to 18 cm within three years due to a new cyst attached to the caudal side of the first cyst. **D** The solid component inside the new cyst (arrowhead) showed enhancement on diffusion-weighted imaging

A 39-year-old female patient was referred to our institution for further investigation of a pancreatic cyst detected by abdominal ultrasonography during her health checkup. Contrast-enhanced abdominal computed tomography revealed a left intraperitoneal multilocular tumor measuring 14 cm (Fig. 1A). Magnetic resonance imaging revealed a smooth mass with homogeneous contents in the pancreatic tail (Fig. 1B). Endoscopic ultrasonography demonstrated a cystic lesion in the pancreatic tail, which was primarily anechoic, with some peripheral stone-like structures and a hyperechoic fluid–fluid level. Communication with the pancreatic duct was unclear. The patient was diagnosed with a benign pancreatic tumor and was followed up every six months.

After 3 years, the tumor showed remarkable progression. Follow-up magnetic resonance imaging revealed a multilocular cystic mass measuring 18 cm (Fig. 1C). A 10-cm cystic mass with solid components was hyperintense on diffusion-weighted imaging (Fig. 1D). The serum carcinoembryonic antigen level was < 18.0 ng/ml, and serum carbohydrate antigen 19–9 was 28 U/ml. Based on these findings, LDP was performed for possible malignancy.

**Fig. 2:**
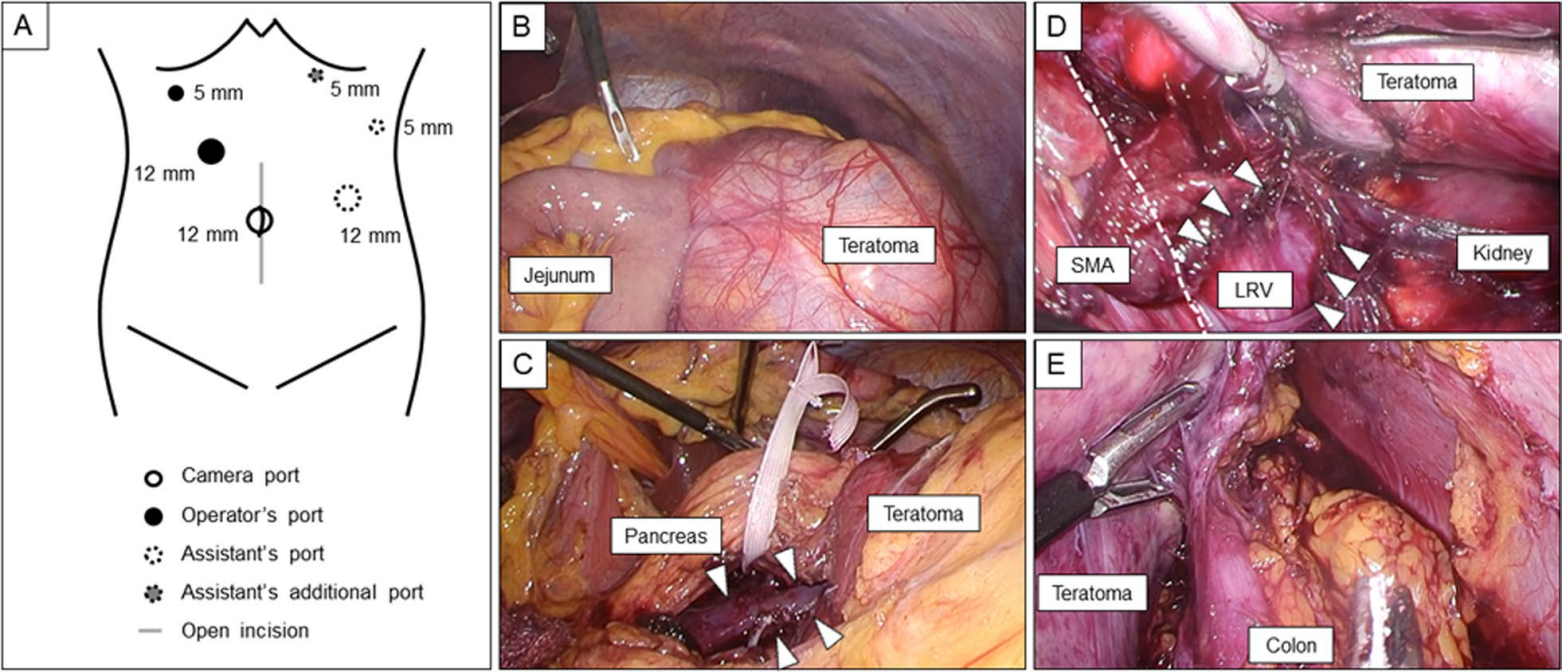
Intraoperative findings. **A** Port placement. **B** A voluminous, subcapsular, elastic, and multilocular cyst was revealed under the transverse colon. **C** Following the detachment of the pancreas from the splenic vein (arrowhead) and artery, the pancreas was transected using a linear stapler along the tape. **D** The adhesiolysis of the tumor from the retroperitoneum required detachment of the superior mesenteric artery (SMA, dash line), left renal vein (LRV, arrowhead), left adrenal gland, and left kidney. **E** Following the detachment of the tumor from the transverse colon, laparoscopic distal pancreatectomy was completed

The first 12-mm trocar was inserted at the umbilicus using open method. After pneumoperitoneum, other four trocars were inserted (Fig. 2A). Laparoscopic examination revealed a voluminous, subcapsular, elastic, and multilocular cyst under the transverse colon (Fig. 2B). The capsule appeared solid enough to withstand retraction of the endoscopic forceps without rupturing. The tumor was considered to originate from the pancreas. It was difficult to dissect the dorsal side of the tumor in the caudal to the cranial direction because of the tumor size. After the splenic artery and vein were divided, the pancreas was transected at the left side of the superior mesenteric vein using a linear stapler with a polyglycolic acid sheet (Fig. 2C). Furthermore, an additional port in the left upper quadrant facilitated tumor elevation and dissection of the dorsal layer of the giant tumor from the retroperitoneum, proximal to the distal region. The tumor compressed the left renal vein because of its size. Following the detachment of the tumor from the superior mesenteric artery, left renal vein, left adrenal gland, left kidney, and transverse colon, LDP was completed (Fig. 2D, E). The operative time and total intraoperative blood and ascites loss were 371 min and 600 ml, respectively.

**Fig. 3:**
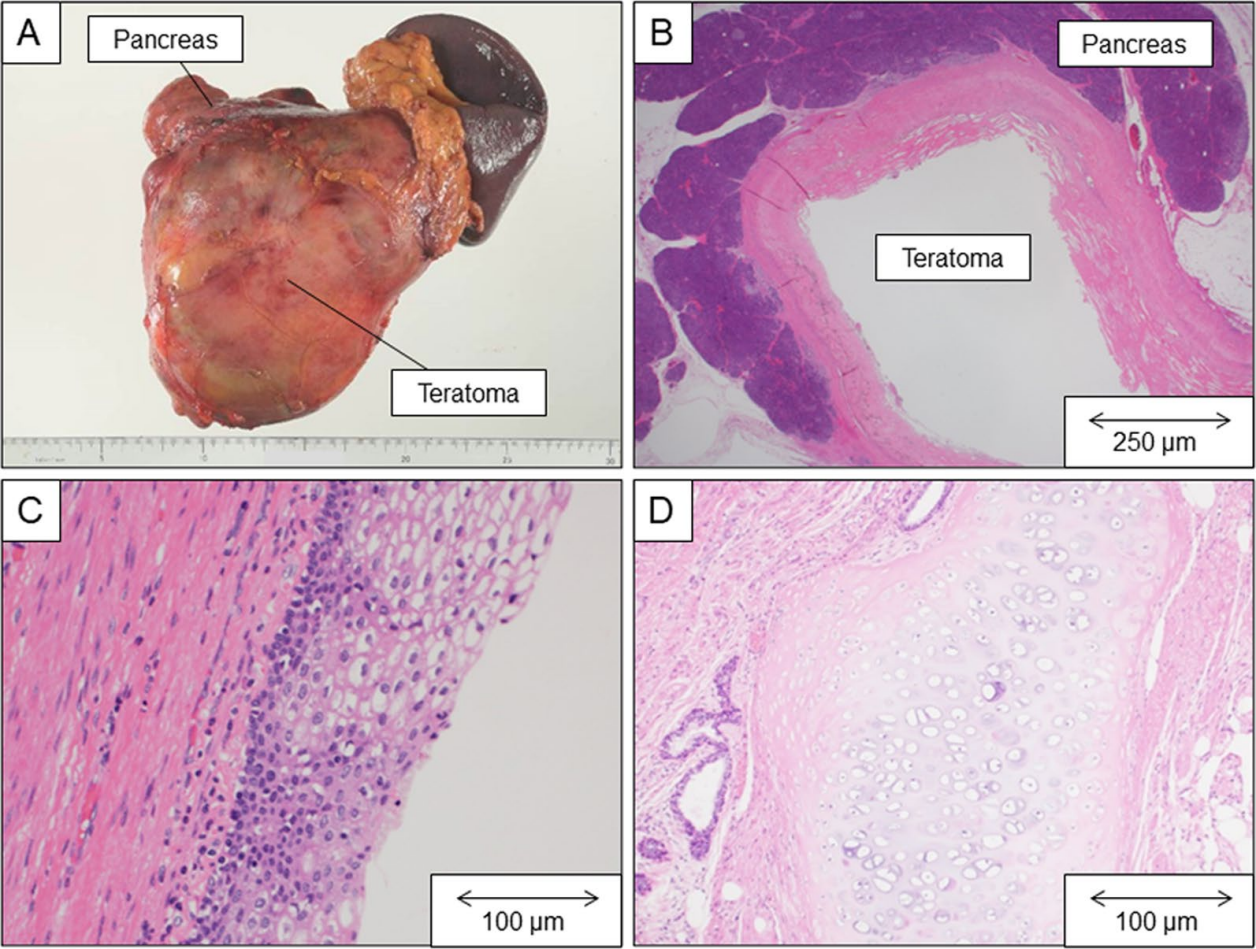
Pathological examinations of the pancreas tumor. This surgical specimen is an 18-cm pancreatic tail cyst with granular sebaceous material in its lumen (**A**). Microscopic observation (hematoxylin and eosin staining) showing the tumor attached to the pancreatic tissue (**B**). The cyst lumen is lined with stratified squamous epithelium (**C**) and cartilage (**D**)

Macroscopic examination revealed an 18-cm multiloculated pancreatic tail cyst with granular sebaceous material inside its lumen (Fig. 3A). Histopathological analysis revealed that the lumen of the cyst at the pancreatic tail (Fig. 3B) was covered with various tissues, including mucinous, ciliated, and stratified squamous epithelium (Fig. 3C); cartilage (Fig. 3D); salivary gland-like organization; muscle; and bulbil. The presence of tissue components in the three germ cell layers led to the diagnosis of mature cystic teratoma of the pancreas.

The postoperative course was uneventful, except for a pancreatic leak, which was well controlled by antibiotics, and the patient was discharged on postoperative day 13. The patient was followed up six months later without signs of recurrence.

## Discussion

Mature cystic teratomas are germ cell-originated, benign, slow-growing tumors composed of mature differentiated elements derived from multiple embryonic layers, also known as dermoid cysts [6]. The pancreas is an extremely rare site of presentation, with only five dozen previously reported clinical cases, none of which involved malignant degeneration. [7, 8]. Despite their benign nature and low risk of malignant transformation, as expected from previous studies on mature ovarian cystic teratomas, preoperative diagnosis is difficult for several reasons [9]. Clinical presentations are nonspecific even in symptomatic cases, including various gastrointestinal complaints, and serum tumor marker levels are mostly negative or otherwise slightly elevated [1, 9–11]. The appearance on radiological and sonographic imaging varies because of the different proportions of tissue components in the cyst [11]. Therefore, complete surgical resection remains the standard of care for diagnostic and curative treatments [7]. In the present case, the preoperative differential diagnosis included an epidermoid or lymphoepithelial cyst owing to the solid component; however, possible malignancy could not be ruled out because of its considerable size.

Because it is slow-growing, pancreatic cystic teratomas tend to present already relatively large, ranging from 2.2 to 22 cm [2, 7]. In most cases, resection is performed by laparotomy, and only three case reports have been published on mature cystic teratomas of the pancreas that underwent minimally invasive surgery (Table 1) [3, 4, 12]. Although LDP may lengthen the operative time compared with open distal pancreatectomy, it has been shown to reduce blood loss, time to functional recovery, and initial hospital stay [13–15]. In a study of five patients with giant pancreatic cystic neoplasms, LDP was shown to be feasible and offered less pain, no wound infections, early return of bowel activity, early return of oral intake and early discharge, which supports LDP as a treatment for a giant cystic teratoma, as in the present case [16]. This study and other reports on large pancreatic lesions have discussed the feasibility and surgical modifications of the laparoscopic approach [17, 18]. The essential steps for a feasible LDP include recognition, dissection of the splenic vessels, and transection of the pancreatic neck, which enables medial-to-lateral mobilization of the pancreas. Previously reported surgical tips for LDP of giant pancreatic cystic lesions include shifting the operative ports, gastric retraction, and aspiration before retrieval. We inserted an additional port in the upper left quadrant after dissecting the pancreatic neck, which enabled better operability of the surgical forceps and instruments. The roll-up technique to retract the stomach ensures wider exposure of the lesser sac [19]. Despite reports of post-resection aspiration using wound protectors and double-balloon catheters after bagging the resected specimen, we could not adopt this method due to fear of intra-abdominal spillage of cystic contents. Considering the limited number of previous studies, more successful surgical modifications are required.

**Table 1:** Clinical characteristics of patients with teratoma of the pancreas who underwent laparoscopic resection

Case	Age	Sex	Complaint	Size	Surgery	Surgical refinements	Complications	Postoperative hospital stays	Recurrence	Outcome	References
	(years)			(cm)				(days)		(months)	
1	54	M	None	7.5	LSPDP	n.d.	None	8	None	Alive, 12	[3]
2	53	M	None	4.4	LDP	n.d.	None	7	None	n.d.	[4]
3	72	M	None	4.2	LDP	Clockwise technique	None	4	None	Alive, 12	[12]
Our case	42	F	None	18	LDP	Additional port	Pancreatic leak	13	None	Alive, 6	–

F, female; LDP, laparoscopic distal pancreatectomy; LSPDP, laparoscopic spleen preserving distal pancreatectomy; M, male; n.d., no data

To our knowledge, the present case is the largest cyst to be resected using a complete laparoscopic approach without intraoperative rupture. With the trend of LDP being implemented worldwide, it should be considered in patients with pancreatic cystic lesions, regardless of size.

## Conclusion

Here, we report a case of a giant mature cystic teratoma of the pancreas that was successfully resected using complete LDP. With some surgical refinements, LDP can be considered in cases of large cystic lesions of the pancreatic body or tail.

## Data Availability

Data sharing is unavailable due to patient privacy concerns but are available from the corresponding author on reasonable request.

## Abbreviation

LDP: Laparoscopic distal pancreatectomy
